# The Expression of Bone Morphogenetic Protein 2 and Matrix Metalloproteinase 2 through Retinoic Acid Receptor Beta Induced by All-Trans Retinoic Acid in Cultured ARPE-19 Cells

**DOI:** 10.1371/journal.pone.0150831

**Published:** 2016-03-11

**Authors:** Zhenya Gao, Lijun Huo, Dongmei Cui, Xiao Yang, Junwen Zeng

**Affiliations:** 1 State Key Laboratory of Ophthalmology, Zhongshan Ophthalmic Center, SunYat-sen University, No.54 South Xianlie Road, Guangzhou, 510060, P. R. China; 2 The First Affiliated Hospital, Sun Yat-sen University, No.58 Zhong Shan Er Road, Guangzhou 510080, P. R. China; University of Florida, UNITED STATES

## Abstract

**Purpose:**

All-trans retinoic acid (ATRA) plays an important role in ocular development. Previous studies found that retinoic acid could influence the metabolism of scleral remodeling by promoting retinal pigment epithelium (RPE) cells to secrete secondary signaling factors. The purpose of this study was to investigate whether retinoic acid affected secretion of bone morphogenetic protein 2 (BMP-2) and matrix metalloproteinase 2 (MMP-2) and to explore the signaling pathway of retinoic acid in cultured acute retinal pigment epithelial 19 (ARPE-19) cells.

**Methods:**

The effects of ATRA (concentrations from 10^−9^ to 10^−5^ mol/l) on the expression of retinoic acid receptors (RARs) in ARPE-19 cells were examined at the mRNA and protein levels using reverse transcription-polymerase chain reaction (RT-PCR) and western blot assay, respectively. The effects of treating ARPE-19 cells with ATRA concentrations ranging from 10^−9^ to 10^−5^ mol/l for 24 h and 48 h or with 10^-6^mol/l ATRA at different times ranging from 6h to 72h were assessed using real-time quantitative PCR (qPCR) and enzyme-linked immunosorbent assay (ELISA). The contribution of RARβ-induced activation of ARPE-19 cells was confirmed using LE135, an antagonist of RARβ.

**Results:**

RARβ mRNA levels significantly increased in the ARPE-19 cells treated with ATRA for 24h and 48h. These increases in RARβ mRNA levels were dose dependent (at concentrations of 10^−9^ to 10^−5^ mol/l) with a maximum effect observed at 10^−6^ mol/l. There were no significant changes in the mRNA levels of RARα and RARγ. Western blot assay revealed that RARβ protein levels were increased significantly in a time-dependent manner in ARPE-19 cells treated with 10^−6^ mol/l ATRA from 12 h to 72 h, with a marked increase observed at 24 h and 48 h. The upregulation of RARβ and the ATRA-induced secretion in ARPE-19 cells could be inhibited by the RARβ antagonist LE135.

**Conclusion:**

ATRA induced upregulation of RARβ in ARPE-19 cells and stimulated these cells to secrete BMP-2 and MMP-2.

## Introduction

Myopia has become a major public health problem worldwide, with a prevalence ranging from 20–30% in North American and European populations to 80–90% in East Asian populations [1–4]. Recent studies have shown that retinoic acid (RA) can be synthesized and secreted by the retina and choroid and function as a biochemical signal [5], that plays an important role in the development and progression of axial myopia [6, 7].

RA is a metabolic product of retinol, the active from of vitamin A, and interacts with two distinct types of intracellular proteins. In the cytoplasm, RA interacts with cellular retinoic-acid-binding proteins (CRABPs), which are primarily involved in the storage, intracellular transport and metabolism of RA [8]. In the nucleus, RA interacts mainly with two families of nuclear retinoid receptor proteins: retinoic acid receptors (RARs) and retinoid “X” receptors (RXRs). Both RARs and RXRs have α, β and γ subtypes with different expression patterns in different cells and tissues [9]. RA exerts its biological functions primarily by binding to and activating specific nuclear receptors [10, 11].

In our previous studies, we proved that ATRA and the retinoid signaling pathway contribute to inhibition of human sclera fibroblast (HSF) proliferation through RARβ [12]. An increasing number of studies have found that RA can influence the scleral extracellular matrix and subsequently increase the size of the eye by stimulating retinal pigment epithelium (RPE) cells to secrete secondary signaling factors that signal the direction of ocular growth [6, 7, 13]. The purpose of the present study was to investigate whether RA affected secretion of bone morphogenetic protein 2 (BMP-2) and matrix metalloproteinase 2 (MMP-2) and to explore the signaling pathway of RA in cultured acute retinal pigment epithelial 19 (ARPE-19) cells

## Materials and Methods

### Ethics statement

No animal or protected plants were involved in this study, and the ecosystem was not compromised by the collection of experimental samples. This study did not harm the natural environment or the health of humans.

### Cell Culture and Cell Treatment

ARPE-19 cells were kindly provided by Prof. Shaochong Zhang (Zhongshan Ophthalmic Center, Sun Yat-sen University, China) who purchased it from the American Type Culture Collection (ATCC). Cells were routinely cultured in Dulbecco's Modified Eagle medium/Ham's Nutrient Mixture F12 (1:1) (DMEM/F12, Gibco, Grand Island, NY) supplemented with 10% fetal bovine serum (FBS, Gibco, Australia), penicillin (100 i.u./ml) and streptomycin (100μg/ml) (Invitrogen, Carlsbad, CA). Then, cells were incubated at 37°C in a humidified incubator containing 5% CO_2_. The medium was changed every two to three days. The cells were trypsinized for 2 minutes in an incubator with 0.25% trypsin /EDTA (Gibco) solution and subcultured at a split ratio of 1:4–6 in a 25-mm^2^ plastic bottle (Corning Ltd., Lowell, MA) to achieve a heavy primary monolayer. The cells were cultured in 60-mm dishes (Corning) to 70–80% confluence for extracting proteins for western blot analysis, Cells were cultured in six-well plates (Corning) for RT-PCR or real-time PCR analysis.

ATRA (Sigma-Aldrich, USA) and LE135 (Tocris Bioscience, UK) were dissolved in dimethyl sulfoxide (DMSO, Sigma-Aldrich, USA) to a stock concentration of 10 mmol/l. ATRA was further diluted to the working concentration with DMEM/F12, before immediate use or until frozen in aliquots at -20°C, avoiding light. The cells preincubated with DMEM/F12 without FBS for 24 hours or were pretreated with 10^−6^ mol/l LE135 for 24 hours. The medium was then changed to ATRA, and the cells were incubated for the appropriate treatment time (0, 6, 12, 24, 48, 72 h) and concentration (0, 10^−9^, 10^−8^, 10^−7^, 10^−6^, 10^−5^ mol/l). The cell culture supernatants were collected and used for ELISA.

### Cell Counting Kit-8 (CCK8) Assay

ARPE-19 cells were plated in 96-well plates at a density of 5,000 cells per well with 100 μl of complete culture medium. After preincubation for 24 hours in DMEM/F12 with 0.1% FBS, the cells were transfected with 10 μl of ATRA at various concentrations (0, 10^−9^, 10^−8^, 10^−7^, 10^−6^, 10^−5^ mol/l). Wells containing only culture medium served as blanks. After the plates were incubated for 24 or 48 h, the supernatant was removed, and 100 μl of DMEM /F12 containing 10 μl of CCK8 (Dojindo, Kumamoto, Japan) was added to each well. The plates were then incubated for another 1–4 hours at 37°C. The absorbance was measured at 450 nm using a microplate reader (PowerWave XS Microplate Spectrophotometer, Bio-Tek, USA). All experiments were repeated independently at least five times.

### Total RNA Isolation, Reverse Transcription, and Real-Time PCR

Total RNA was isolated using a PureLinkTM RNA Mini Kit (Invitrogen, USA) according to the manufacturer’s instructions. The quantity and concentration of total RNA were assessed at 260 and 280 nm using Synergy H1 Hybrid Reader (BioTek, USA). Total RNA was stored at -80°C. A 1 μg sample of total RNA was subsequently reverse transcribed for first-strand cDNA synthesis using the PrimeScript RT Master Mix Perfect Real Time kit (TaKaRa, Japan) under following the conditions: 37°C for 15 min, immediately at 85°C for 5 sec, and then maintained at 4°C. The cDNA was diluted 1:1 and then subjected to RT-PCR and real-time PCR.

The oligonucleotide primer sequences used in RT-PCR to investigate transcript expression are listed in Table 1. The primers were designed using NCBI Pick Primers and were synthesized by Invitrogen^TM^ Custom DNA Oligos (Life Technologies, USA). Each PCR was performed in a 25 μl solution containing 1 μl (10μmol /L) of each forward and reverse primer, 12.5 μl of Premix Ex Taq DNA polymerase (TaKaRa, Japan), and 1 μl of reverse transcription reaction products. Amplification was conducted for 30 cycles in a professional thermocycler (Biometra, Germany). Each cycle consisted of denaturation for 45 sec at 94°C, annealing for 30 sec at 60°C, and extension for 60 sec at 72°C. PCR amplification of GAPDH1 was performed in parallel as an internal control. The amplified products were analyzed via electrophoresis on a 1% agarose gel (Invitrogen) containing ethidium bromide and then photographed under ultraviolet light illumination. A standard DNA ladder was used as a size marker. All experiments were performed in triplicate. All RT-PCR products were compared to GAPDH1 cDNA products from the corresponding samples, and all band intensities were evaluated using densitometry.

**Table 1:** Primer sequences used in RT-PCR.

Gene	GenBank number	Primer sequences	Product size(bp)
**RARα**	NM_001024809.3	Forward: 5’-CTGCCAGTACTGCCGACTGC-3’ Reverse: 5’-ACGTTGTTCTGAGCTGTTGTTCGTA-3’	235
**RARβ**	NM_016152	Forward: 5’-CAGGACCTTGACCAACCGACAA-3’ Reverse: 5’-GAGAGGTAGCATTGATCCAGGA-3’	210
**RARγ **	NM_001042728.1	Forward: 5’-CTGCCAGTACTGCCGGCTAC-3’ Reverse: 5’-TCTGCACTGGAGTTCGTGGTATACT-3’	228
**RXRα**	NM_002957	Forward: 5’-GGATCCCACACTTCTCAG-3’ Reverse: 5’-GAGTCAGGGTTAAAGAGGAC-3’	286
**RXRβ**	NM_021976	Forward:5’-AGTACTGCCGCTATCAGAA-3’ Reverse: 5’-GTTAGTCACAGGGTCATTTG-3’	242
**RXRγ**	NM_006917	Forward: 5’-CTACAGATACCCCAGTGA-3’ Reverse: 5’-GGGTAGTTCATGTTTCCAAT-3’	249
**GAPDH1**	NM_002046.3	Forward: 5’-GCTCAGACATGGGGAAGGT-3’ Reverse: 5’-GTGGTGCAGGAGGCATTGCTGA-3’	474

The primer pairs used in real-time PCR assays to investigated transcript expression are listed in Table 2. Real-time PCR was performed using a LightCycler 480 SYBR Green I Master and Roche LightCycler 480 real-time system. The real-time PCR reaction mixtures contained a total volume of 20 μl, which included 12.5 μl of SYBR Green I Master Mix, 1 μl of forward and reverse primers (10 μmol/l), 2 μl cDNA, and 6 μl ddH_2_O. The real-time PCR programs were as follows: one cycle of 95°C for 5 min, followed by 45 cycles of 95°C for 10 s, annealing at 60°C for 10 s, and 72°C for 10 s. To complete the protocol, a melting curve was constructed using the following program: 95°C for 5 s, 65°C for 60 s, and 97°C while continuously collecting fluorescence signals. Three independent experiments were performed for each sample. The relative gene expression levels were determined using the 2^-ΔΔ^Ct method.

**Table 2:** Primer sequences used in qRT-PCR.

Gene	GenBank number	Primer sequences	Product size(bp)
**RARβ**	NM_016152	Forward: 5’- CGTGGAGTTTGCTAAACGTCT-3’ Reverse: 5’-TGGTGTCTTGTTCT GGGGTAT-3’	134
**BMP-2**	NM_001200.2	Forward: 5’- CCCGACACTGAGACGCTGTTC-3’ Reverse: 5’- GGGGAAGCAGCAACGCTAGAA-3’	243
**MMP-2**	NM_004530.4	Forward: 5’- AGATCTTCTTCTTCAAGGACCGGT -3’ Reverse: 5’- GGCTGGTCAGTGGCTTGGGGTA -3’	225
**GAPDH2**	NM_002046.3	Forward: 5’- GATTCCACCCATGGCAAATT-3’ Reverse: 5’- TCTCGCTCCTGGAAGATGGT-3’	94

### Western Blot Analysis

Cells were washed three times with PBS at 4°C and then lysed in 150 μl cold 1X RIPA lysis buffer (CST, USA) supplemented with protease inhibitor cocktail tablets (Roche, Germany). The protein concentration was determined using a BCA Protein Assay Kit (Thermo Scientific, USA). Protein extracts were separated using 10% sodium dodecyl sulfate-polyacrylamide gels (SDS-PAGE), blotted on polyvinylidene difluoride (PVDF) membranes (Roche) and blocked with Tris-buffered saline with 0.05% Tween 20 (TBST) containing 5% nonfat milk. The membranes were then exposed to the following primary antibodies: rabbit polyclonal RARβ (Santa Cruz, USA) (1:700), rabbit polyclonal GAPDH (Proteintech, USA) (1:4000). Primary antibodies were diluted in TBST with 5% nonfat milk, and incubated overnight at 4°C. The membranes were washed in TBST, and then incubated with a secondary goat anti-rabbit lgG antibody (CST) conjugated with horseradish peroxidase at 1:3000 for one hour at room temperature. Finally, chemoluminescence signals were visualized with an ECL (Millipore, USA) imager, and analyzed using BIO-RAD Quantity One Imaging software (Bio-Rad, USA).

### Enzyme-linked immunosorbent assay (ELISA)

ELISA kits for BMP-2 and MMP-2 were purchased from RayBiotech (Atlanta, USA). ELISAs were performed according to the manufacturer’s instructions. Experiments were performed in triplicate for concordant results.

### Statistical analysis

The quantitative data are expressed as the mean ± SD. The statistical significance of inter-group differences was determined with one-way analysis of variance (ANOVA) followed by Bonferroni’s multiple comparison test. Bartlett’s test was used to determine the homogeneity of variance. P values less than 0.05 were considered statistically significant (*P<0.05, **P<0.001).

## Results

### Effect of ATRA on the survival of ARPE-19 cells

**Fig 1:**
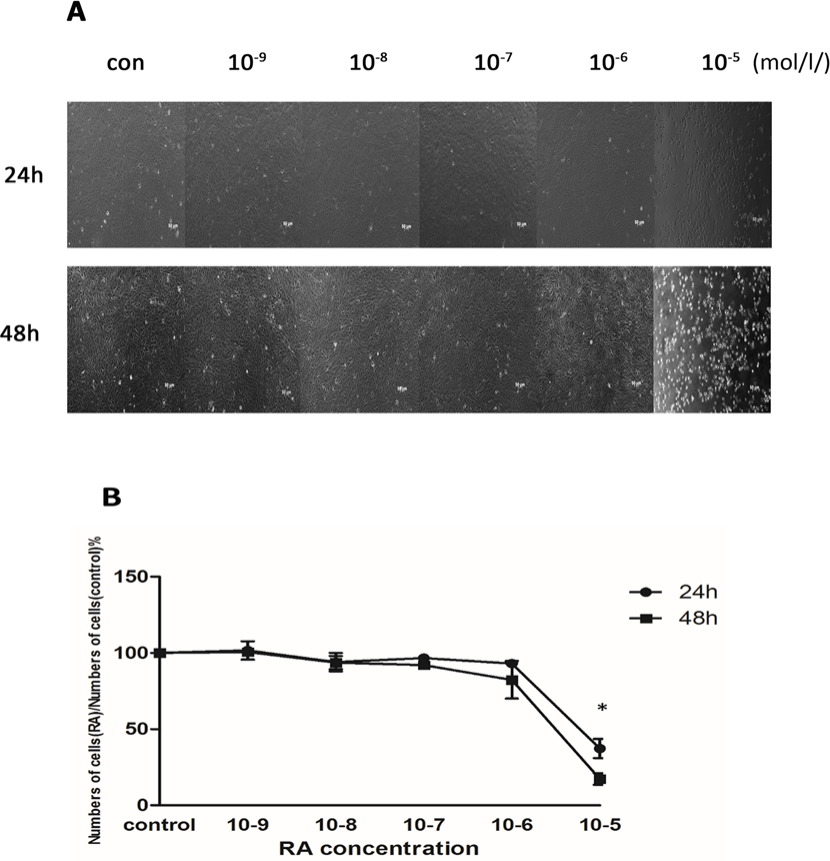
The Effect of ATRA on survival in ARPE-19 cells. ATRA caused a low level of apoptosis (<10%) at concentrations from 10^−9^ to 10^−6^ mol/l, but the cell morphology remained normal. The cells were inhibited at the highest concentration of ATRA treatment 10^−5^ mol/l (Fig 1A). At both 24 h and 48 h, the cell proliferation was inhibited by ATRA in a dose-dependent manner. A small reduction (<10%) in the number of cells also occurred with a lower ATRA concentration of 10^−6^ mol/l, but these changes were not statistically significant (P>0.05). When the ATRA concentration was 10^−5^ mol/l, the cell survival rate was below 50% at both time points (Fig 1B).

ARPE-19 cells were treated with increasing concentrations of ATRA, from 10^−9^ to 10^−5^mol/l, to determine whether ATRA affected their survival. We found that ATRA caused a low level of apoptosis (< 10%) at concentrations between 10^−9^ and 10^-6^mol/l, whereas the cell morphology remained normal. The cells were inhibited at 10^-5^mol/l, the highest concentration of ATRA tested (Fig 1A). Then, the effect of ATRA on ARPE-19 cells survival was evaluated by incubating them with different concentrations of ATRA for 24h and 48 h using a CCK-8 (Fig 1B). At both 24 h and 48 h, ATRA inhibited cell survival in a dose- dependent manner. A small reduction (<10%) in cell number also occurred with ATRA concentrations less than 10^−6^ mol/l, but the changes were not statistically significant (P>0.05). The cell survival rate was less than 50% at both time points when the concentration of ATRA was 10^-5^mol/l.

### Effect of ATRA on the expression of RARβmRNA and protein

**Fig 2:**
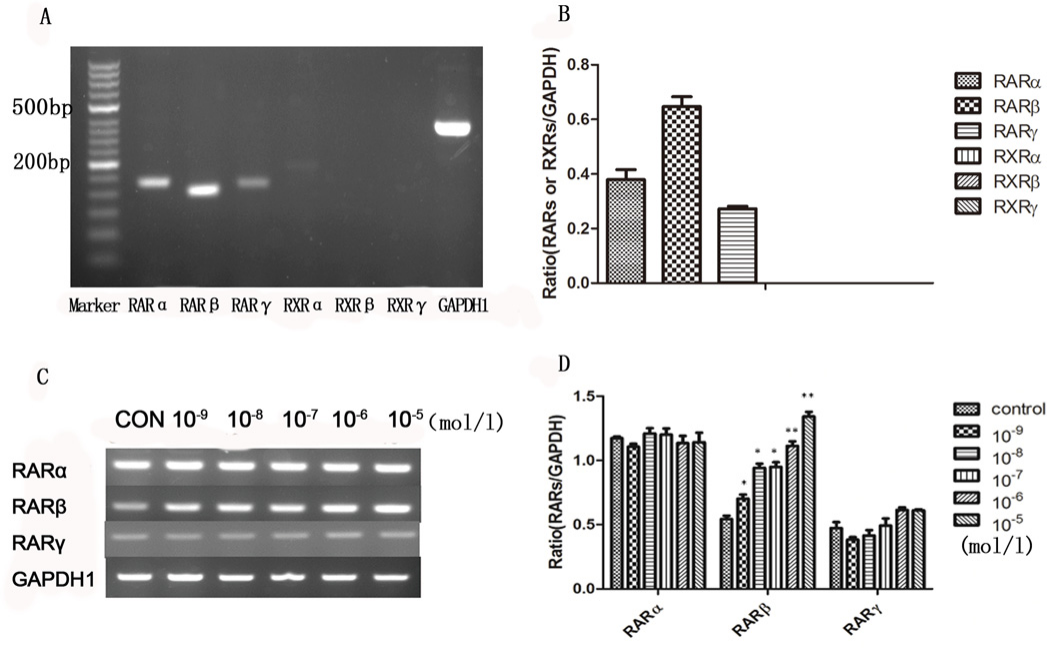
RARs and RXRs expressions in ARPE-19 cells. RARs mRNA was strongly expressed in ARPE-19 cells, whereas RXRs expression was not detected (Fig 2A and 2B). RARβ mRNA levels was increased after ATRA treatment when compared with controls. Expression of RARβ mRNA was dose dependent with a maximal effect observed with 10^−5^ mol/l ATRA. In contrast, there were no significant changes in the mRNA levels of RARα and RARγ (P>0.05) (Fig 2C and Fig 2D).

**Fig 3:**
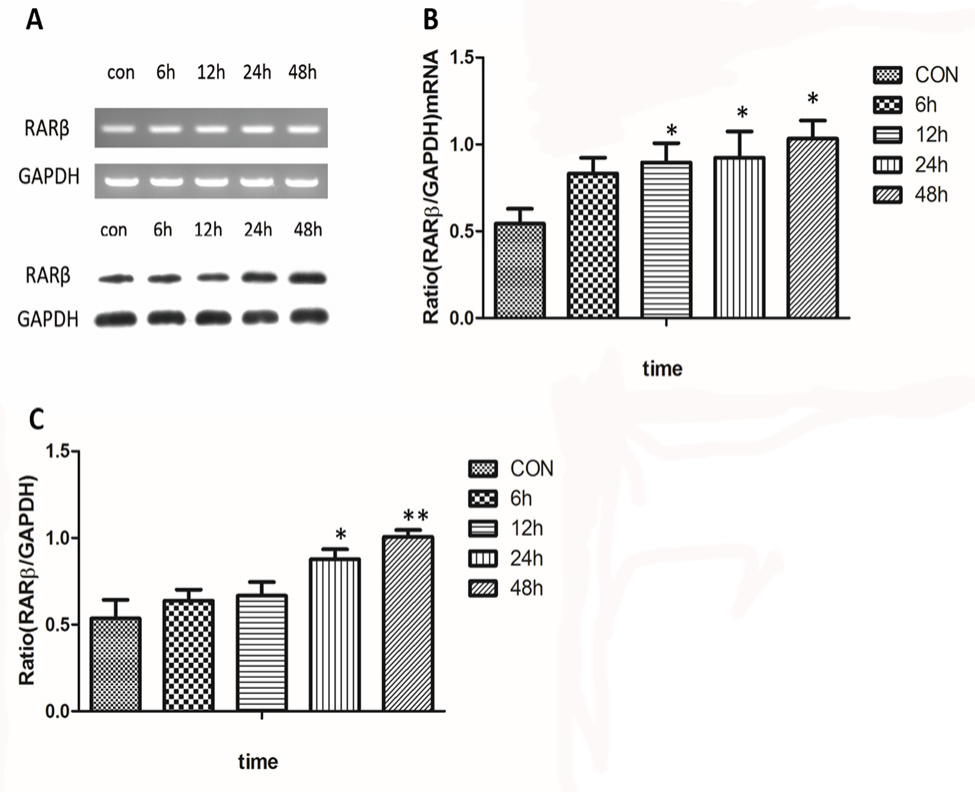
Time-related changes in expression of RARβ mRNA and protein. A significant increase in the RARβ band intensity was detected for both mRNA (Fig 3A and 3B) and protein (Fig 3A and 3C) at 24 h and 48 h after treatment with 10^−6^ mol/l ATRA (P<0.05).

First, the expression of RARs and RXRs in ARPE-19 cells was detected at the genetic level using RT-PCR. We found that RARs were strongly expressed in ARPE-19 cells, whereas RXRs expression was not detected (Fig 2A and 2B). Next, ARPE-19 cells were treated with different concentration of ATRA (from 10^−9^ to 10^−5^ mol/l) for 24 hours identify the RA receptor through which ATRA activated the signaling pathway in ARPE-19 cells. In the treated group, RARβ mRNA levels increased after incubation with ATRA, when compared with the control group, and this increase was dose dependent with a maximal effect at 10^−5^ mol/l. In contrast, there were no significant changes in the RARα and RARγ mRNA levels (Fig 2C and 2D). Finally, to quantify RARβ expression over time, RARβ mRNA and protein were isolated at 6, 12, 24, and 48 h with 10^−6^ mol/l ATRA. A significant increase in the RARβ band intensity was detected after the addition of ATRA at 24 h and 48 h (Fig 3).

### Up-regulation of BMP-2 and MMP-2 expression in ARPE-19 cells treated with ATRA

**Fig 4:**
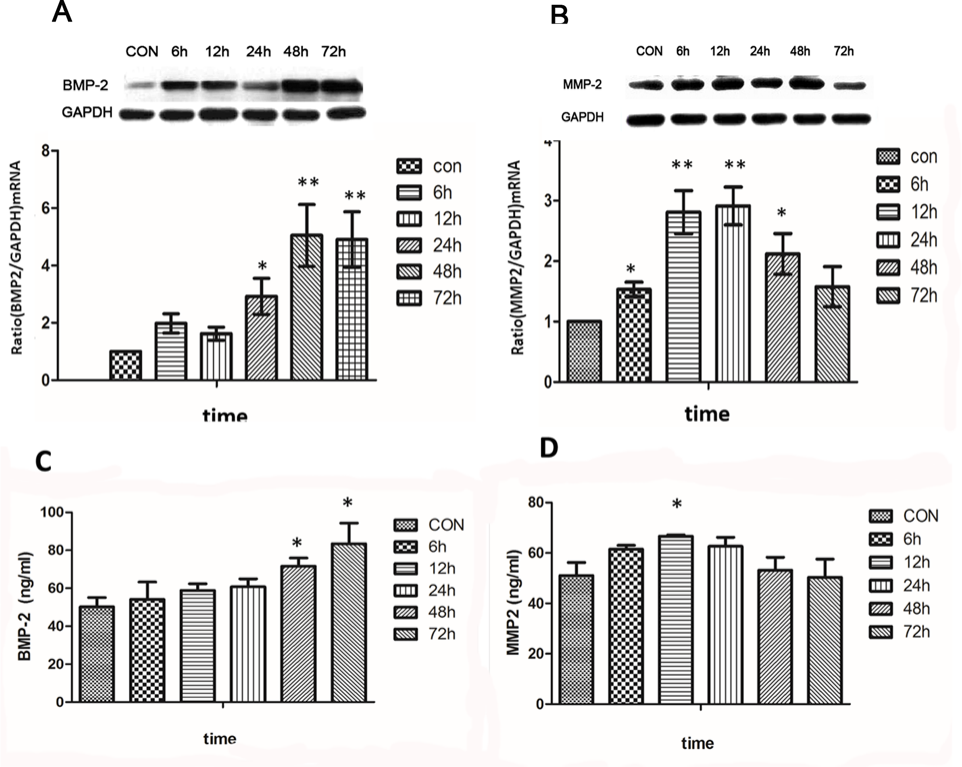
BMP-2 and MMP-2 expression in ARPE-19 cells at different times points following ATRA treatment. ATRA at 10^−6^ mol/l upregulated the expression of BMP-2 mRNA in ARPE-19 cells, and this effect was time dependent. There were no significant changes in BMP-2 mRNA in ARPE-19 cells after incubation with ATRA for 12 h, but the mRNA levels of BMP-2 were significantly increased after treatment with 10^−6^ mol/l ATRA for 48 h and 72 h (p<0.001) (Fig 4A). However, the marked expression of MMP-2 mRNA was upregulated in ARPE-19 cells after incubation with ATRA at 10^−6^ mol/l for 6 h, peaked at 12 h and 24 h (P<0.001), but was then down regulated to normal levels at 72 h (P>0.05) (Fig 4B). The expression levels of BMP-2 and MMP-2 protein were similar to the levels of those mRNA in ARPE-19 cells (Fig 4A and 4B). Cell supernatant from the cells treated with ATRA concentrations of 10^−6^ mol/l were analyzed by ELISA and compared with controls (medium with 0.1% DMSO). ATRA also upregulated the BMP-2 protein level in a time dependent manner. BMP-2 protein expression was significantly increased after the cells were treated with 10^−6^ mol/l ATRA at both 48 h and 72 h (p<0.05) (Fig 4C). The MMP-2 protein level was similar to its mRNA level and was significantly increased at 12 h (P<0.05) (Fig 4D).

BMP-2 and MMP-2 expression were detected in ARPE-19 cells after treatment with 10^-6^mol/l ATRA for 6, 12, 24, 48, and 72 h using real-time PCR, western blot and ELISA. ATRA at 10^−6^ mol/l upregulated expression of BMP-2 mRNA and protein in ARPE-19 cells in time-dependent manner. There were no significant changes in BMP-2 mRNA in ARPE-19 cells after incubation with ATRA for 12 h (P>0.05), but BMP-2 mRNA levels were significantly increased after incubation with 10^−6^ mol/l ATRA for 48 h and 72 h (p<0.001) (Fig 4A). However, the marked expression of MMP-2 mRNA was upregulated after being incubated with ATRA for 6 h, and peaked at 12 h and 24 h (P<0.001), but was downregulated to normal levels at 72h (Fig 4B). The expression levels of BMP-2 and MMP-2 protein were similar to the levels of those mRNA in ARPE-19 cells (Fig 4A and 4B). Cell supernatants were collected and analyzed using ELISA. We found that ATRA also upregulated BMP-2 protein levels in a time-dependent manner. BMP-2 protein expression was significantly increased after the cells were treated with 10^−6^ mol/l ATRA for 48 h and 72 h (p<0.05) (Fig 4C). The levels of MMP-2 protein and mRNA was similar, and were significantly increased at 12 h (P<0.05) (Fig 4D).

### ATRA-induced BMP-2 and MMP-2 expression are inhibited by the RARβ antagonist LE135

**Fig 5:**
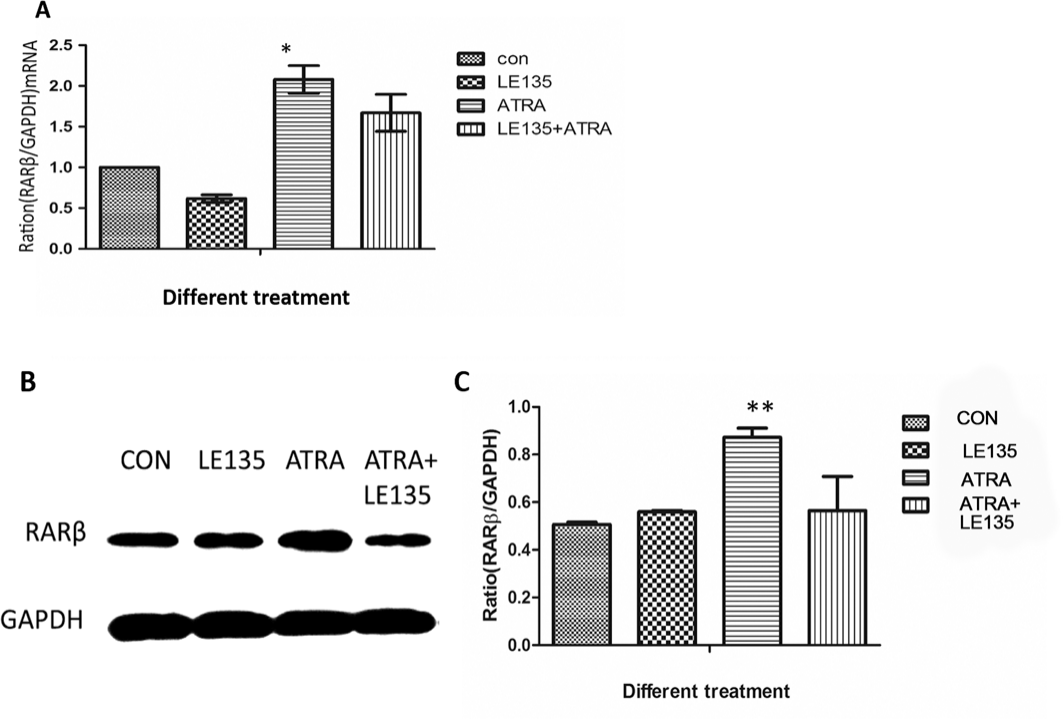
LE135 could block the pathway of RARβ in ARPE-19 cells. Compared to the control, LE135 (10^-6^mol/l) could block the ATRA-induced increase in RARβ expression at either the mRNA (P<0.05) (Fig 5A) or the protein level (P<0.001) (Fig 5B and 5C) at 48 h.

**Fig 6:**
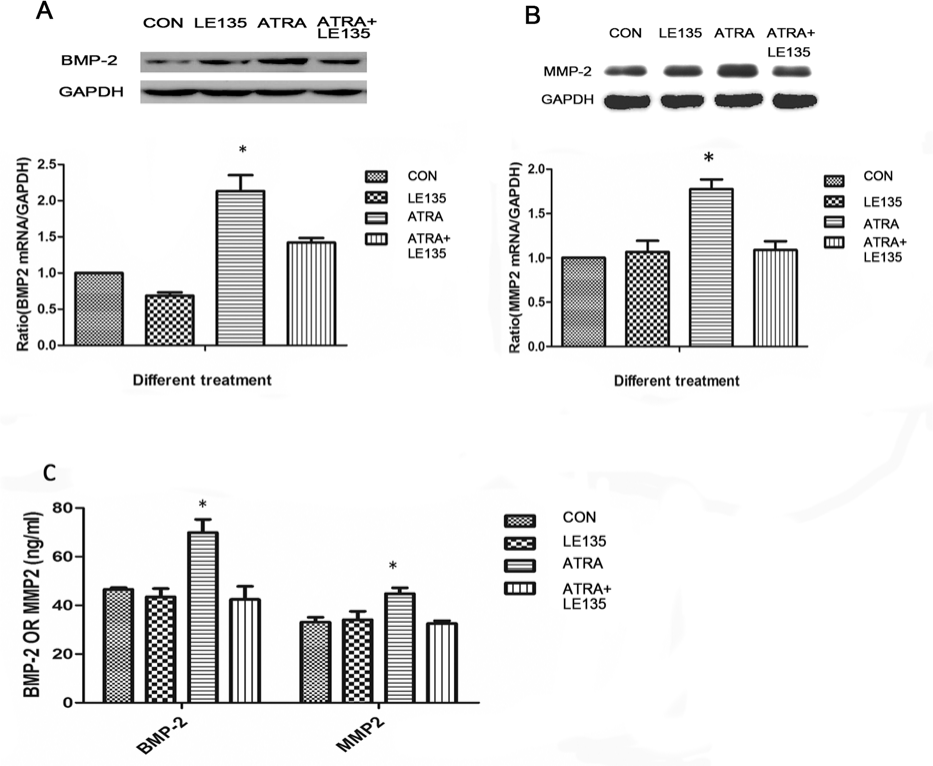
ATRA-induced BMP-2 and MMP-2 expression were inhibited by RARβ. The increased expression of BMP-2 and MMP-2 induced by ATRA were almost completely blocked by pre-treatment of the ARPE19 cells with LE135. ATRA at 10^−6^ mol/l upregulated the expression of BMP-2 and MMP-2 on both mRNA and protein levels in ARPE-19 cells (P<0.05), but there were no significant changes after incubation with LE135 (10^-6^mol/l) or ATRA (10^-6^mol/l) added to LE135 (10^-6^mol/l) (Fig 6A, 6B and 6C).

The correlation among BMP-2, MMP-2 and RARβ in ARPE-19 cells treated with ATRA prompted us to investigate whether the increased expression of BMP-2 and MMP-2 might be secondary to an increased expression of RARβ. Therefore, we pre-treated the ARPE-19 cells with the RARβ antagonist LE135 before ATRA treatment. As shown in Fig 5, compared to the control, LE135 almost completely blocked the increased RARβ expression induced by ATRA both in the mRNA level and the protein level at 48h. The ATRA-induced increases in BMP-2 and MMP-2 expression were blocked by pre-treatment with LE135 (Fig 6).

## Discussion

In the present study, ATRA caused a low level of apoptosis (< 10%) at concentrations from 10^−9^ to10^-6^ mol/l, but cell morphology remained normal. These findings might be explained by the low concentrations of ATRA we used in this study. We found that the survival of ARPE-19 cells was inhibited by ATRA in a dose-dependent manner, especially with an ATRA concentration of 10^−5^ mol/l and when the cells exposed to ATRA exhibited characteristic morphological changes. This result is consistent with those in our previous report [14]. We then further investigated the expression of RARs and RXRs in the ARPE-19 cells, which revealed that the ARPE-19 cells specifically expressed RARs mRNA; RXRs mRNA expression was not detected. Exposure to ATRA appeared to increase RARβ mRNA levels in a dose-dependent manner, with a maximum effect at 10^−6^ mol/l, and this increase was consistently matched with the increase in RARβ protein levels. After treatment with ATRA, up-regulation of RARβ protein levels could be detected at 6 h, were significantly increased after 24 h (P <0.05), and showed the largest increase at 72 h (P <0.001). Our finding that ATRA exposure led to an up-regulation of RARβ is in line with the results of previous experiments that investigated other cell types [15]. RA exerted its influence on mammalian cells by binding to and activating the transcriptional activity of specific nuclear receptors [9, 16]. Furthermore, evidence from several studies indicates that RARβ can play a central role in regulating cell proliferation [10, 17, 18].

Previous studies clarified that ATRA could inhibit the growth of some cells by blocking at the G1 phase of the cell cycle, and ATRA exerted these effects primarily by modulating the expression of genes involved in G1-checkpoint regulation [19, 20]. Those studies are in accord with our previous findings in HSF cells exposed to RA [21]. Many studies had shown that RA might contribute to the occurrence and progression of axial myopia. Endogenous levels of retinal-RA and choroidal/scleral-RA were clearly increased in form-deprivation myopia (FDM) in the chick and guinea pig [13, 22]. Furthermore, intravitreal injection of disulfiram, an inhibitor of RA synthesis, reduced the degree of FDM [13]. Visual deprivation also produced an up-regulation of the expression of RARβ in the sclera of chicks [23] and retina of guinea pigs [24]. When the eyeball is rapidly elongated, as occurs in FD, the scleral thickness decreases [13]. However, previous studies indicated that factors originating in the retina that are related to the occurrence of axial myopia could not pass through the RPE-choroid without interruption to influence the sclera directly [25, 26]. Thus, the RPE, which is interposed between the retina and the choroid, is likely critical in relaying retinal growth signals to the choroid and sclera. The “local control theory of the retina” theory states that these retinal factors (such as RA) activate RPE cells and stimulate their release of secondary signaling factors (such as TGF-β, BMP-2, MMP-2) that regulate ocular growth and subsequently lead to axial myopia [27]. Thus, RPE cells may play a crucial role between the sclera and messengers from the retina.

In this study, we observed that the mRNA and protein levels of BMP-2 and MMP-2 in ARPE-19 cells were upregulated after treatment with 10^−6^ mol/l ATRA, and this upregulation could be blocked by LE135, a specific antagonist of RARβ. These results would confirm the theory described above.

The outer coating of the eyeball, the human sclera, consists of several types of collagens (e.g., type I, type III, type V), aggrecan and scleral fibroblast cells. As axial myopia progresses, the biomechanical properties of the sclera are altered, especially the posterior sclera. In the posterior sclera remodeling, it was characterized by changing the scleral extracellular matrix (ECM), such as decreasing collagen I, increasing levels of MMP-2 [28]. In previous studies, BMP-2, which belongs to the TGF-β superfamily, was detected in the human and guinea pig sclera. In addition, BMP-2 was reduced in the posterior sclera of FDM guinea pigs, which further indicates that BMP-2 influenced ECM and scleral reconstruction in progress of human myopia [29, 30]. Scleral tissue is degraded during remodeling, which is partially regulated by MMPs and TIMPs [31]. MMP-2, an important member of the MMPs family, is a protein that is initially secreted as an inactive proenzyme and became an active enzyme by self-cleavage. MMP-2 expression was confirmed in the human sclera, and it was increased in the sclera of eyes with deprivation-induced axial myopia when compared with the control eyes in chicks and tree shrews [32, 33]. In our previous study, we also found that HSF cells showed a decreased MMP-2 expression after incubation with BMP-2 [29]. Therefore, BMP-2 and MMP-2 are both important growth factors that influence HSF cells and are possibly involved in remodeling the sclera. Our findings also suggested that BMP-2 might play a key role in scleral remodeling during the progress of axial myopia. BMP-2 promoted the proliferation of HSF cells and altered expression of MMP-2 and TIMP-2[34].

Although we cannot concluded from the current findings that ATRA directly affects RPE cells, our observations certainly support the plausible hypothesis that ATRA was increased in retina in axial myopia, where it activates RPE cells, and promotes their release of scleral remodeling factors (such BMP-2, MMP-2). These changes would subsequently disequilibrate sclera remodeling. As a result, the vitreous chamber would become elongated excessively and enable the progression of axial myopia. Previous studies revealed that ARPE-19 cells are similar to native RPE cells, but not identical [35, 36], so we will confirm the results in native RPE cells in the future.

The findings of this study suggested that ATRA can promote BMP-2 and MMP-2 expression in ARPE-19 cells through RARβ. This signaling pathway may play a critical role in the progression of axial myopia and is a potential candidate for myopia management.

## References

[1] Morgan I., Rose K., How genetic is school myopia? Prog Retin Eye Res, 2005 24(1): p. 1–38. 10.1016/j.preteyeres.2004.06.004 . 15555525

[2] Vitale S., Sperduto R.D., Ferris F.R., Increased prevalence of myopia in the United States between 1971–1972 and 1999–2004. Arch Ophthalmol, 2009 127(12): p. 1632–9. 10.1001/archophthalmol.2009.303 . 20008719

[3] Lin L.L., Shih Y F, Hsiao C K, Chen C J., Prevalence of myopia in Taiwanese schoolchildren: 1983 to 2000. Ann Acad Med Singapore, 2004 33(1): p. 27–33. . 15008558

[4] Pan C.W., Ramamurthy D., Saw S.M., Worldwide prevalence and risk factors for myopia. Ophthalmic Physiol Opt, 2012 32(1): p. 3–16. 10.1111/j.1475-1313.2011.00884.x . 22150586

[5] Mertz J.R., Wallman J., Choroidal retinoic acid synthesis: a possible mediator between refractive error and compensatory eye growth. Exp Eye Res, 2000 70(4): p. 519–27. 10.1006/exer.1999.0813 10866000

[6] McFadden S.A., Howlett M H, Mertz J R, Wallman J., Acute effects of dietary retinoic acid on ocular components in the growing chick. Exp Eye Res, 2006 83(4): p. 949–61. 10.1016/j.exer.2006.05.002 . 16797531

[7] Troilo D., Nickla D L, Mertz J R, Summers, Rada JA., Change in the synthesis rates of ocular retinoic acid and scleral glycosaminoglycan during experimentally altered eye growth in marmosets. Invest Ophthalmol Vis Sci, 2006 47(5): p. 1768–77. 10.1167/iovs.05-0298 . 16638980 PMC1892188

[8] Si S.P., Tsou H C., Lee X., Peacocke M., Effect of cellular senescence and retinoic acid on the expression of cellular retinoic acid binding proteins in skin fibroblasts. Exp Cell Res, 1995 219(1): p. 243–8. 10.1006/excr.1995.1224 . 7628539

[9] Chambon P., A decade of molecular biology of retinoic acid receptors. FASEB J, 1996 10(9): p. 940–54. . 8801176

[10] Geisen C., Denk C., Gremm B., Baust C., Karger A. Bollag W., et al, High-level expression of the retinoic acid receptor beta gene in normal cells of the uterine cervix is regulated by the retinoic acid receptor alpha and is abnormally down-regulated in cervical carcinoma cells. Cancer Res, 1997 57(8): p. 1460–7. . 9108446

[11] Pavan B., Biondi C., Dalpiaz A., Nuclear retinoic acid receptor beta as a tool in chemoprevention trials. Curr Med Chem, 2006 13(29): p. 3553–63. . 17168722 10.2174/092986706779026183

[12] Huo L., Cui D., Yang X., Gao Z., Trier K., Zeng J., All-trans retinoic acid modulates mitogen-activated protein kinase pathway activation in human scleral fibroblasts through retinoic acid receptor beta. Mol Vis, 2013 19: p. 1795–803. . 23946634 PMC3742120

[13] McFadden S.A., Howlett M.H. and Mertz J.R., Retinoic acid signals the direction of ocular elongation in the guinea pig eye. Vision Res, 2004 44(7): p. 643–53. . 14751549 10.1016/j.visres.2003.11.002

[14] Li C., McFadden S.A., Morgan I., Cui D., Hu J., Wan W., et al, All-trans retinoic acid regulates the expression of the extracellular matrix protein fibulin-1 in the guinea pig sclera and human scleral fibroblasts. Mol Vis, 2010 16: p. 689–97. . 20405022 PMC2855729

[15] Xu X.C., Liu X., Tahara E., Lippman S M., Lotan R., Expression and up-regulation of retinoic acid receptor-beta is associated with retinoid sensitivity and colony formation in esophageal cancer cell lines. Cancer Res, 1999 59(10): p. 2477–83. . 10344761

[16] Mangelsdorf D.J., Evans R.M., The RXR heterodimers and orphan receptors. Cell, 1995 83(6): p. 841–50. . 8521508 10.1016/0092-8674(95)90200-7

[17] Ju J., Wang N, Wang X, Chen F., A novel all-trans retinoic acid derivative inhibits proliferation and induces differentiation of human gastric carcinoma xenografts via up-regulating retinoic acid receptor beta. Am J Transl Res, 2015 7(5): p. 856–65. . 26175847 PMC4494137

[18] Cheung B., Hocker J. E., Smith S. A., Norris M. D., Haber M., Marshall G. M., Favorable prognostic significance of high-level retinoic acid receptor beta expression in neuroblastoma mediated by effects on cell cycle regulation. Oncogene, 1998 17(6): p. 751–9. 10.1038/sj.onc.1201982 . 9715277

[19] Qiao J., Paul P., Lee S., Qiao L., Josifi E., Tiao J. R., et al, PI3K/AKT and ERK regulate retinoic acid-induced neuroblastoma cellular differentiation. Biochem Biophys Res Commun, 2012 424(3): p. 421–6. 10.1016/j.bbrc.2012.06.125 . 22766505 PMC3668681

[20] Hu X.T., Zuckerman K.S., Role of cell cycle regulatory molecules in retinoic acid- and vitamin D3-induced differentiation of acute myeloid leukaemia cells. Cell Prolif, 2014 10.1111/cpr.12100 . 24646031 PMC6496847

[21] Shirakami Y., Gottesman M.E., Blaner W.S., Diethylnitrosamine-induced hepatocarcinogenesis is suppressed in lecithin:retinol acyltransferase-deficient mice primarily through retinoid actions immediately after carcinogen administration. Carcinogenesis, 2012 33(2): p. 268–74. 10.1093/carcin/bgr275 . 22116467 PMC3271263

[22] Seko Y., Shimokawa H., Tokoro T., In vivo and in vitro association of retinoic acid with form-deprivation myopia in the chick. Exp Eye Res, 1996 63(4): p. 443–52. 10.1006/exer.1996.0134 . 8944551

[23] Bitzer M., Feldkaemper M., Schaeffel F., Visually induced changes in components of the retinoic acid system in fundal layers of the chick. Exp Eye Res, 2000 70(1): p. 97–106. 10.1006/exer.1999.0762 . 10644425

[24] Huang J., Qu X.M., Chu R.Y., Research on retinoic acid signals in retina of guinea pig eyes with different monochromatic illumination. Zhonghua Yan Ke Za Zhi, 2011 47(10): p. 938–43. . 22321506

[25] Rymer J., Wildsoet C.F., The role of the retinal pigment epithelium in eye growth regulation and myopia: a review. Vis Neurosci, 2005 22(3): p. 251–61. 10.1017/S0952523805223015 . 16079001

[26] Mao J.F., Liu S.Z., Dou X.Q., Retinoic acid metabolic change in retina and choroid of the guinea pig with lens-induced myopia. Int J Ophthalmol, 2012 5(6): p. 670–4. 10.3980/j.issn.2222-3959.2012.06.04 . 23275899 PMC3530806

[27] Heberlein U., Moses K., Mechanisms of Drosophila retinal morphogenesis: the virtues of being progressive. Cell, 1995 81(7): p. 987–90. . 7600585 10.1016/s0092-8674(05)80003-0

[28] McBrien N.A., Gentle A., Role of the sclera in the development and pathological complications of myopia. Prog Retin Eye Res, 2003 22(3): p. 307–38. . 12852489 10.1016/s1350-9462(02)00063-0

[29] Hu J., Cui D., Yang X., Wang S., Hu S., Li C., et al, Bone morphogenetic protein-2: a potential regulator in scleral remodeling. Mol Vis, 2008 14: p. 2373–80. . 19098993 PMC2605409

[30] Wang Q., Zhao G., Xing S., Zhang L., Yang X., Role of bone morphogenetic proteins in form-deprivation myopia sclera. Mol Vis, 2011 17: p. 647–57. . 21403850 PMC3056124

[31] Shelton L., Rada J.S., Effects of cyclic mechanical stretch on extracellular matrix synthesis by human scleral fibroblasts. Exp Eye Res, 2007 84(2): p. 314–22. 10.1016/j.exer.2006.10.004 . 17123515 PMC2583333

[32] Guggenheim J.A., McBrien N.A., Form-deprivation myopia induces activation of scleral matrix metalloproteinase-2 in tree shrew. Invest Ophthalmol Vis Sci, 1996 37(7): p. 1380–95. . 8641841

[33] Rada J.A., Brenza H.L., Increased latent gelatinase activity in the sclera of visually deprived chicks. Invest Ophthalmol Vis Sci, 1995 36(8): p. 1555–65. . 7601636

[34] Gao Z.Y., Huo L.J., Cui D.M., Yang X., Wan W.J., Zeng J.W., Distribution of bone morphogenetic protein receptors in human scleral fibroblasts cultured in vitro and human sclera. Int J Ophthalmol, 2012 5(6): p. 661–6. 10.3980/j.issn.2222-3959.2012.06.02 . 23275897 PMC3530804

[35] Ablonczy Z., Dahrouj M., Tang P.H., Liu Y., Sambamurti K., Marmorstein A.D., et al, Human retinal pigment epithelium cells as functional models for the RPE in vivo. Invest Ophthalmol Vis Sci, 2011 52(12): p. 8614–20. 10.1167/iovs.11-8021 . 21960553 PMC3208409

[36] Strunnikova N.V., Maminishkis A., Barb J.J., Wang F., Zhi C., Sergeev Y., et al, Transcriptome analysis and molecular signature of human retinal pigment epithelium. Hum Mol Genet, 2010 19(12): p. 2468–86. 10.1093/hmg/ddq129 . 20360305 PMC2876890

